# Evaluation of the single and combined therapeutic effects of individually manufactured earplug therapy in patients with myogenous temporomandibular disorders: A randomized controlled clinical trial

**DOI:** 10.1007/s00784-024-05956-0

**Published:** 2024-10-01

**Authors:** Merve Benli, Monica J. Cayouette

**Affiliations:** Department of Reconstructive and Rehabilitation Sciences, College of Dental Medicine, Medical University of South Carolina, 173 Ashley Ave Room: BSB 505, Charleston, SC USA

**Keywords:** Earplug, Occlusal splint, Pain measurement, Mouth opening, Myogenous temporomandibular disorders

## Abstract

**Objective:**

To evaluate the effect of individually manufactured earplug therapy on pain intensity (PI), symptom severity (SS), and maximum mouth opening (MMO), in patients with myogenous temporomandibular disorders (TMD).

**Methods:**

One-hundred-twenty patients were randomly allocated to six groups: Groups EP (earplug), OS (occlusal splint), EX (exercise), EPO (earplug with occlusal splint), EPE (earplug with exercise), and C (control). Outcomes were PI (assessed with a visual analog scale (VAS)), SS (assessed with the modified Symptom Severity Index Questionnaire (mSSI)), and MMO (evaluated with a digital caliper). Measurements were performed at T0 (before the therapy), T1 (1-month follow-up), and T2 (3-month follow-up). Data were analyzed using one-way analysis of variance (ANOVA), Tukey’s HSD, and chi-square tests (alpha = 0.05).

**Results:**

At T1 and T2, the greatest VAS and mSSI reduction was detected for the groups EPE (VAS = 5.3 ± 1.05, 3.3 ± 0.7; mSSI = 38.2 ± 2.27, 43.6 ± 3.94) and EPO (VAS = 5.2 ± 0.91, 3.2 ± 0.78; mSSI = 36.3 ± 3.97, 42.2 ± 3.19), respectively (*p* < 0.05). At T1, occlusal splint groups (groups OS (34.8 ± 2.97 mm) and EPO (33.8 ± 3.49 mm)) gave the highest MMO values, while T2 values did not constitute a significant difference with T1 (*p* > 0.05).

**Conclusions:**

The short-term use of combined earplug therapy resulted in a decrease in both PI and SS. Improvement in MMO in participants using occlusal splints was observed in the 1st month and was maintained through the 3rd month.

**Clinical Relevance:**

Earplug therapy can be applied as a complementary therapy to occlusal splint and exercise treatments to decrease PI and SS in patients with myogenous TMD. To achieve functional recovery such as MMO, its combined use with splints should be taken into consideration by clinicians.

## Introduction

Temporomandibular disorder (TMD), with a prevalence of up to 12%, is a common disease in society [1, 2]. In recent years, TMD treatment trends have moved towards multi-modal and multi-disciplinary treatment in parallel with other chronic musculoskeletal disorders [3]. Many therapeutic procedures have been proposed to treat TMD, such as splint therapy, pharmacotherapy, occlusal adjustment, physical therapy, surgery, acupuncture, massage therapy, biofeedback, stress management, and cognitive behavioral therapy [4]. These strategies often recommend reversible and less invasive therapies, with the gold standard for myogenous TMD treatment being occlusal splint therapy [4, 5].

Although the most widely accepted current therapeutic approach for TMD is occlusal (intraoral stabilization) devices/splints, its exact mechanism of action is poorly understood. Irreversible complications may occur if used for a long time [4, 6]. The e-devices have disadvantages, such as interference with eating and speech, comfort, and aesthetics, which may limit their effect to nighttime use only [7]. There are also reports of clinically significant, irreversible occlusal changes due to occlusal splints [8]. For this reason, different treatment methods have been suggested in the current literature to achieve better clinical results, such as earplugs.

The ear canal is closely related to the temporomandibular joint (TMJ), both physiologically and anatomically [7]. A study conducted on human cadavers revealed that there is a connection between TMJ and the middle ear thanks to the disco malleolar ligament by connecting the TMJ disc to the malleus bone of the middle ear, which lies along the petrotympanic fissure, which explains the ear symptoms in patients with TMD. Another study stated that TMD patients had more otological symptoms than normal individuals [9]. The ear canal does not consist of a rigid tube connected to the middle ear, and its outer 1/3 is highly deformable with movements of the mandible and tongue, which is supported by the finding in a recent study that mandibular movements cause deformations in the ear [10].

Recently, some commercial and custom-made earplugs purporting to treat TMD have been introduced with few published studies, such as TMDs (Ascentia Health Inc, Rockford, IL, USA), later renamed Cerezen [6, 7, 11]. The device consists of a hollow, hard plastic tube that fits into the patient's ear canal, and they have a metal handle so that the patient can easily insert and remove it [6]. There are only two randomized controlled trials in the literature on this subject, and the results obtained with the patient groups evaluated differ [6, 7]. In one of these studies, only patients with myogenous TMD were assessed in terms of pain and mouth opening index, and it was found that earplug treatment was more advantageous than occlusal splint in terms of these parameters [7]. In another study, patients with different types of TMD (arthralgia, myofascial pain, and disc displacement with reduction) were compared regarding pain, symptom severity, and Fricton's craniomandibular index [12]. The study revealed that the earplug group performed significantly better than splint and exercise therapies in all evaluated parameters [6]. In existing studies, earplug therapy has been applied alone, and no attempt has been made to assess the combined effect of this treatment with other methods under the 'complementary treatment concept,' a concept whose popularity for TMD treatment is increasing daily. Thus, the current study aims to fill this gap in the literature.

Considering the information mentioned above, this study aims to evaluate the short-term effects of earplugs on pain intensity (PI), symptom severity (SS), and maximum mouth opening (MMO), both alone and as a ‘combined treatment.’ The hypothesis tested was that combined earplug treatment would produce more advantageous results in reducing pain and increasing mouth opening than other TMD treatments.

## Materials and methods

### Study design

The present six-arm parallel design randomized clinical trial was performed on subjects from the Istanbul University, Faculty of Dentistry, Istanbul-Turkey. Intervention and follow-up sessions were applied between April 2023 and December 2023. The study was approved by the Ethics Committee of the Istanbul University, Faculty of Medicine (protocol number 2022/13; ClinicalTrials Identifier: NCT05362877), and the ethical guidelines of the Declaration of Helsinki were followed. Patients gave written informed consent to participate in this study after fully explaining the procedures. The study fulfilled the guidelines of the Consolidated Standards of Reporting Trials (CONSORT) guidelines to enhance the data quality [13].

## Study population

Subjects were recruited from the Istanbul University Faculty of Dentistry patient list. Diagnostic Criteria for Temporomandibular Disorders (DC/TMD) defined myogenous TMD patients [14]. For this purpose, axis I of the DC/TMD was administered by a single, trained researcher, and axis II was performed by the patients individually without time restriction. Patients exhibiting symptoms and signs of myogenous TMD were enrolled in this study if they met the following inclusion criteria:

1. Diagnosis of myogenous TMD and pain according to the DC/TMD.
2. Age between 18 and 65 years.
3. Minimum pain intensity of 50 mm on a 100 mm visual analog scale (VAS).
4. Natural posterior occlusion.
  The exclusion criteria were as follows:
  1. History of temporomandibular joint (TMJ) surgery or injection.
  2. Presence of a systemic disorder that could compromise the masticatory system (e.g.,epilepsy, neurological disorders, cerebral palsy, among others),
  3. Any physiotherapeutic treatment of the masticatory system.
  4. Previous TMD treatment more recently than a year.
  5. Major psychosocial problems (axis II screening tools).
  6. Ongoing orthodontic treatment.
  7. Wearing a removable or fixed partial prosthesis.
  8. Inflammatory connective tissue diseases.
  9. Having other types of musculoskeletal pains.
  10. Pregnancy.
  11. Obstructive sleep apnea.
  12. Local skin infection over myofascial region muscles.
  13. Medication use for orofacial pain.
  14. Other symptoms of an orofacial disease (e.g.migraine, toothache).
  15. History of facial trauma.
  16. The unwillingness to assume responsibility for the study.
  17. Alcohol or drug abuse.

### Sample size assumption, allocation, and study groups

The sample size determination was executed based on previous studies, which evaluated ear device systems for treating TMD patients [6, 7]. Power analysis (PS software; Dupont and Plummer, 1997) used power = 0.80 and alpha = 0.05 with a d = 0.76 effect size. The minimum number of samples required for each group was 17, but 129 patients were invited to this study due to potential losses.

Groups were allocated based on a randomized controlled trial, and the random sampling method was performed using a web-based number generator [15]. All files were enumerated and divided into one to five groups consecutively. Patients were randomly assigned to one of the following groups, depending on the type of TMD therapy applied:

- **Group EP:** Test group; earplug; *n* = 20.
- **Group OS**: Test group; hard occlusal splint; *n* = 20.
- **Group EX**: Test group; exercise; *n* = 20.
- **Group EPO:** Test group; earplug with hard occlusal splint; *n* = 20.
- **Group EPE:** Test group; earplug with exercise; *n* = 20.
- **Group C:** Control group; without therapy; *n* = 20.

### Earplug therapy and group interventions

**Fig. 1:**
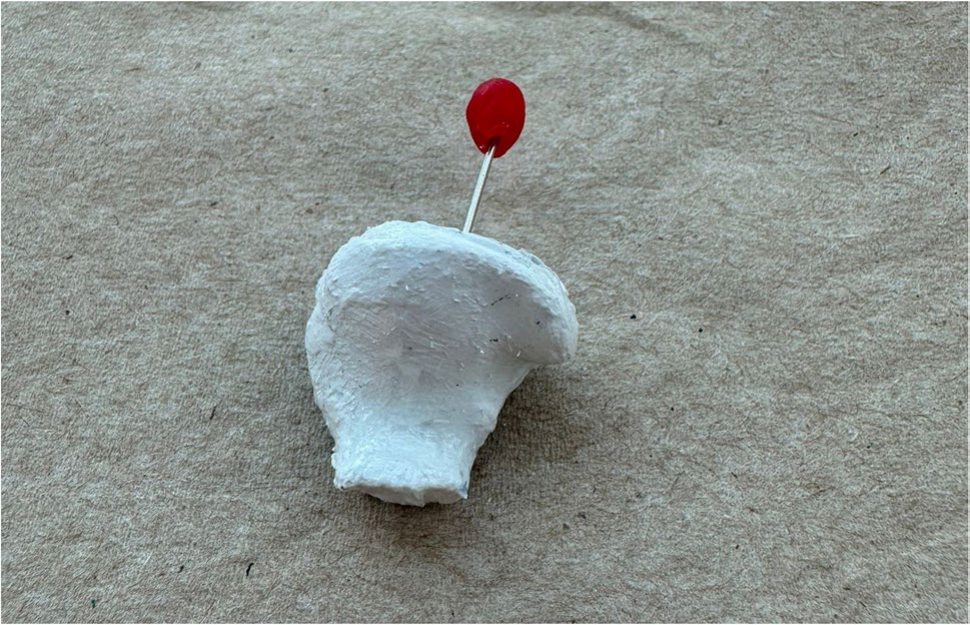
Therapeutic ear plug employed in the groups EP, EPO, and EPE

An ear-nose-throat specialist first accepted patients assigned to the earplug therapy groups (groups EP, EPO, and EPE) to examine ear canal anatomy, remove existing earwax, and ensure that the patient was free of any ear pathology. Then, patients were sent back to the TMJ center to make ear impressions. Before the impression, ear impression pads with traction strings (Otoblock; Amplifon, Milan, Italy) were placed halfway down, just beyond the level of the 2nd bend of the ear canal, to prevent the flow of impression material toward the eardrum. The patient was asked to open their mouth as much as possible to place a mouth prop to help stabilize the lower jaw during the impression-making. Then, the soft vinyl polysiloxane (SiliClone S-50; Westone, Cincinnati, OH, USA) was prepared and mixed into a special syringe, filled into the external auditory canal, and applied into the ear, extending outward and filling the helix bowl and tragus. After the impression material was set, it was slowly removed from the patient's ear, and according to the records obtained from the impressions, the individual earplugs were fabricated from shell material by the same technician (Fotoplast; Dreve Otoplastik GmbH, Unna, Germany) **(**Fig. 1). Each earplug was marked with a red dot for the right and a blue dot for the left. The earplugs were designed to sit flush with the outer edge of the ear canal, were perforated lengthwise to prevent hearing impairment, and had small metal handles added to aid insertion and removal by the patient.

The same dentist performed the insertion and correct positioning of the earplugs. The dentist then instructed patients about insertion/removal, care, and storage. The earplugs were delivered to the patients in a box containing a cleaning brush and one small bottle of lubricating oil (Audinell Natural Lubricant Oil; MG Developpement, Mauguio, France). For patients to gradually get used to the earplugs, participants were recommended to wear the devices for a few hours during the day before using them at night.

In the first week after the devices were delivered, patients were called for a check-up, and it was evaluated whether they had any complaints about the earplugs. The patients' experiences and possible concerns with earplugs were discussed, and whether the wearing mode was correct was checked. In case of any problem, an attempt was made to correct the situation, and if the problem persisted, impressions were retaken for the remanufacturing of the devices. Subsequent recall visits were scheduled for weeks 4 and 12. Subjects in the earplug groups were instructed to wear devices (described previously) as tolerated during the day and night.

**Fig. 2:**
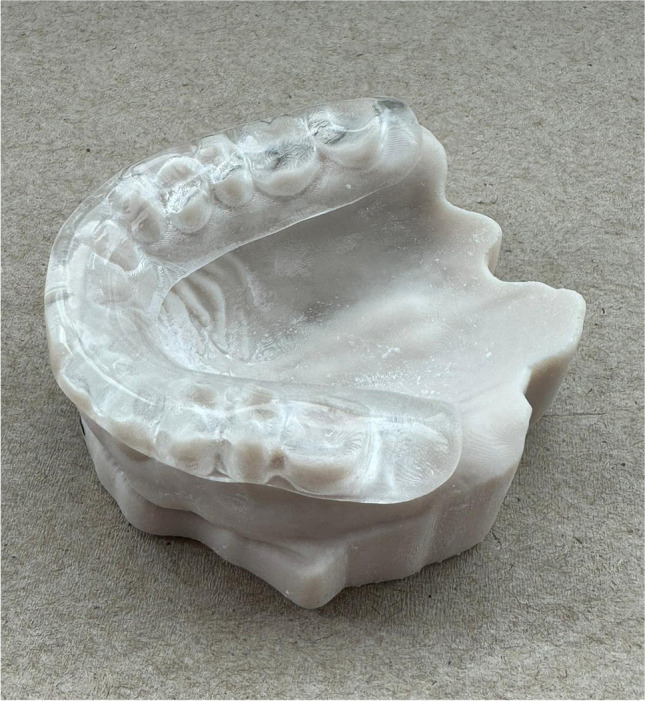
Occlusal splint employed in the groups OS and EPO

For the splint therapy groups (OS and EPO), maxillary and mandibular casts were obtained from the patients to facilitate the fabrication of occlusal splints. These casts were mounted on semi-adjustable articulators at maximum intercuspation (Hanau™ Wide-Vue Articulator; Whip Mix Corporation, KY, USA). The maxillary casts were waxed, and the splints were fabricated from heat-polymerized acrylic resin following the manufacturer's instructions (Trevalon; Dentsply Sirona, NC, USA). Full coverage maxillary splints were manufactured with a thickness of 3 mm in the maxillary and mandibular first molars, and the splint thickness in the anterior region varied between 3–5 mm depending on the occlusal relationship of the patient **(**Fig. 2). Simultaneous and symmetric occlusal contacts were established with canine guidance, and all occlusal splints were finished and polished [16].

The protocol applied in the exercise therapy groups (EX and EPE) was based on the previous study by Kalamir et al. [5]. These exercise sessions included teaching and supervising self-care exercises to ensure proper form during the session and performed by the participant twice daily at home. Exercises designed to stimulate and stretch the TMJ capsule and relax the chewing muscles were as follows: 1) Guided and controlled jaw excursions, 2) Post-isometric stretches (opening), and 3) Post-isometric stretches (lateral deviation) [5].

The control group received no therapy during the study and was told that TMD treatment would be eligible after 12 months of symptom monitoring. This group was blinded to their status as control; however, they were invited to the clinic several times to have all study groups be homogeneous regarding exposure to the dentist. All groups were asked to avoid taking any medications like analgesic drugs, muscle relaxants, tranquilizers, or antidepressants during the research.

## Data collection

A single-blinded researcher in the group allocation collected the data from all the patients by interviewing them face-to-face. Parameters of SS, PI, and MMO were evaluated three times based on the Diagnostic Criteria for Temporomandibular Disorders: Assessment Instruments (DC/TMD); T0, before the intervention; T1, one month; and T2, three months after the selected therapy or therapy combination began.

### PI measurement

The visual analog scale (VAS) was used to measure subjects’ perceived PI [17]. During the measurement, patients were explained that the VAS classification ranged from 0 to 10, with 0 being “no pain” (left), 5 being “moderate pain,” and 10 being “maximum pain imaginable” (right). This scale was applied to compare the PI of the groups between three measurement times (T0, T1, and T2). During the interviews, all patients were told to fill out the VAS by marking the appropriate value, indicating their pain intensity.

### SS measurement

A modified Symptom Severity Index Questionnaire (mSSI) was applied to assess the degree to which patients perceived their TMD as a problem [18]. Within the scope of this index, participants were asked to rate their pain experience by filling in five items (evaluating pain frequency, intensity, duration, discomfort, or difficulty of bearing) for three different locations (temporomandibular joint, masseter, and temple). Possible pain locations evaluated in the index were schematized with a lateral view of the face, head, and jaw. Patients marked one of the 28 bubbles on the optical scanning form as an ordinal scale to represent their pain experience for each of the 15 items. Each scale had a numerical range for scoring, with the leftmost bubble being 0 and the rightmost bubble being 1. All five scales were then averaged to obtain the overall summary score.

### Functional evaluation

MMO was assessed as the "maximal unassisted opening" value based on E4B in the DC/TMD form. The MMO of all groups was measured by adding the maximum inter-incisal distance between the mandibular and maxillary reference teeth (incisors) and the vertical overlap of these teeth to the final value by using a digital caliper (AEK-Tech, Ankara, Turkey). The patients' heads were supported in a neutral position during the measurements. To achieve a stable and reproducible neutral head position, patients were seated in a prepositioned chair according to instructions mentioned in a previous study [4]. A plumb line hanging from the ceiling and parallel to the patient's left arm was used to determine each patient's neutral position. The position where the patient's acromion and ear tragus were divided into two by the plumb line was accepted for assessment. After positioning the head, patients were requested to open their mouths as much as possible to obtain the recorded value. For the reliability of MMO measurements, each participant was made to repeat the mouth-opening movement three times and the average of these values was calculated.

### Statistical analysis

Statistical analysis was performed using standard statistical software (SPSS V23; IBM Armonk, NY, USA) with a significance level of p < 0.05. Conformity to normal distribution was assessed by the Shapiro–Wilk test. Intra-group time comparisons of normally distributed data were analyzed by repeated-measures analysis of variance (ANOVA). The difference between groups over time was analyzed by one-way ANOVA. Multiple comparisons were carried out with Tukey’s HSD. ANOVA was performed to compare quantitative descriptive statistics by groups, and the chi-square test was applied to compare categorical data.

## Results

**Fig. 3:**
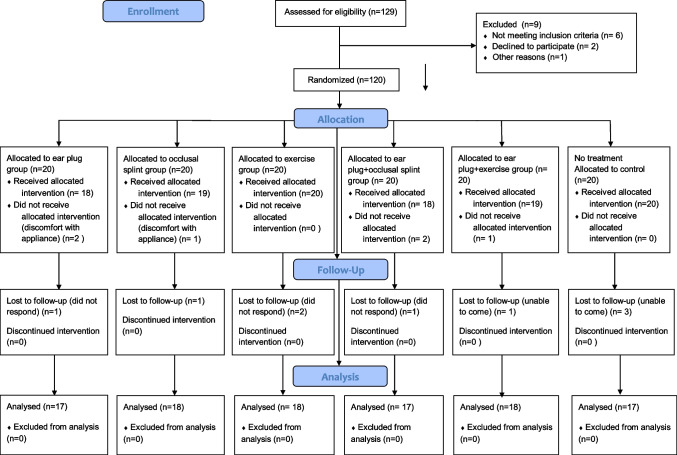
Flowchart of the study

During the research, three patients, 2 in group EP and 1 in group OS withdrew from the study, stating that they were uncomfortable using the devices (earplug and occlusal splint). Therefore, no statement could be made regarding the therapeutic effect in these patients. One hundred-five patients were enrolled in this study, and the overall arrangement of participants was summarized in Fig. 3**.**

Wearing modality, difficulties with the insertion of earplugs, and side effects were evaluated for all participants of the groups EP, EPO, and EPE. Earplugs failed in only one patient regarding retention, and this problem was eliminated by refabricating the devices with the help of new impression records.

A contact point evaluation method determined the overall fit of the occlusal splints for the groups OS and EPO. Static occlusion was evaluated according to the technique mentioned in a previous study, by using an occlusion foil (12 µm thickness, Hanel; Coltene, Langenau, Germany) for each participant [19]. Based on the results, no patient required additional procedures or new splints during the intervals.

### Demographic data

As displayed in the flowchart of this study, 120 out of 129 myogenous TMD patients were selected as eligible and randomly allocated **(**Fig. 3). Fifteen patients (3 from group EP, two from group OS, two from group EX, three from group EPO, two from group EPE, and three from group C) dropped out of the study as they could not come to the clinic or did not respond. Statistical analyses displayed that those who dropped out after randomization were not significantly different from those who completed this study on pretreatment demographics or dependent variables.

The demographic data of the patients is shown in Table 1. The duration of the myogenous TMD ranged from 3 to 5.5 years, and the age ranged between 24 and 45 years. The majority of the participants were female (*p* = 0.83), and weight and height parameters were significantly different between the groups (*p* < 0.05).

**Table 1:** Demographic data of the study groups

Groups							
**Demographic data** (Mean ± SD)	**EP**(N = 17)	**OS**(N = 18)	**EX**(N = 18)	**EPO**(N = 17)	**EPE**(N = 18)	**C**(N = 17)	p*
Age (years)	31.3 ± 7.6	28.9 ± 4.7	32.6 ± 5.9	35.3 ± 7.1	28.8 ± 9.1	29.3 ± 8.7	0.31
Height (cm)	169.5 ± 8.1	155.3 ± 2.7	164.6 ± 7.7	164 ± 5.6	167.6 ± 6.6	172.8 ± 7.1	0
Weight (kg)	69.5 ± 8.1	60.3 ± 9.8	64.6 ± 7.5	63.6 ± 5.1	66.2 ± 6.5	75.2 ± 6.3	0.0007
Duration of disease (years)	3.4 ± 2	3.4 ± 1.7	3 ± 1.6	3 ± 1.7	3.5 ± 2	2.4 ± 1.07	0.72
**Gender**	**Frequency** (%)	**Frequency** (%)	**Frequency** (%)	**Frequency** (%)	**Frequency** (%)	**Frequency** (%)	p**
Male	5 (29.4%)	7(38.9%)	8 (44.4%)	6 (35.3%)	5 (27.8%)	6 (35.3%)	0.83
Female	12 (70.6%)	11 (61.1%)	10 (55.6%)	11(64.7%)	13 (72.2%)	11 (64.7%)	
**Marital status**							
Married	7 (41.2%)	4 (22.2%)	6 (33.3%)	7 (41.2%)	7 (38.9%)	5 (29.4%)	0.72
Single	10 (58.8%)	14(77.8%)	12 (66.6%)	10 (58.8%)	11 (61.1%)	12 (70.6%)	
**Residence**							
Rural	1 (5.9%)	2(11.1%)	0(0%)	1(5.9%)	1(5.5%)	1(5.9%)	0.95
Urban	16(94.1%)	16(88.9%)	18(100%)	16(94.1%)	17(94.5%)	16(94.1%)	

(E Earplug group, S Occlusal splint group, EX Exercise group, E-S Earplug + Occlusal Splint, E-EX Earplug + Exercise group,* and ** significant difference)

### Device use and exercise compliance by the patients

The average times that the groups spent wearing earplugs and occlusal splints and performing exercises at times T1 and T2 were measured as follows:

Earplug = EP (18.8 h/day), EPO (20.3 h/day), and EPE (21.1 h/day),

Occlusal splint = OS (9.8 h/day), and EPO (10.1 h/day),

Exercise = EX (8.3 recurrence), and EPE (8.8 recurrence).

### VAS

**Fig. 4:**
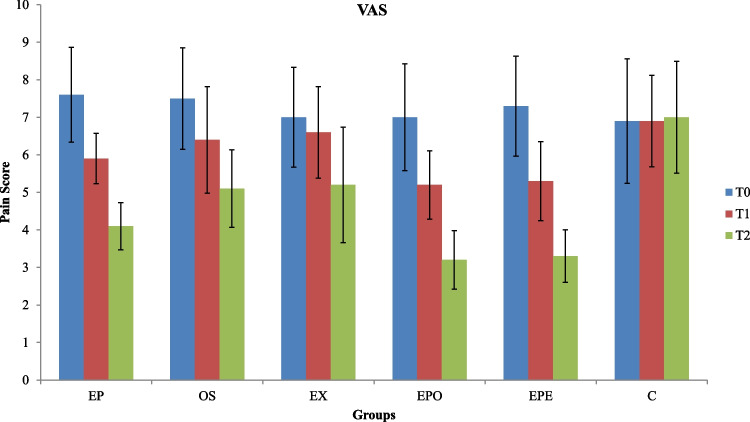
Visual Analogue Scale (VAS) values of the groups from baseline(T0) to 1(T1) and 3 months(T2) (EP, Earplug group; OS, Occlusal splint group; EX, Exercise group; EPO, Earplug + Occlusal Splint, EPE, Earplug + Exercise group;C, Control group; *, significant difference)

Earplug groups combined with exercise and occlusal splints (groups EPE and EPO) demonstrated a statistically significant reduction in PI based on VAS from T0 to T2 (*p* = 0.003) **(**Fig. 4).

At T0, all groups had the same median value (p = 0.856). At T1, all test groups showed improvement by reducing VAS scores, and the most significant reduction was detected for the groups EPE (5.3 ± 1.05) and EPO (5.2 ± 0.91) (*p* = 0.0023); however, the differences between these two groups did not rise to the level of statistical difference (p > 0.05). While the decrease in VAS achieved by these two groups was followed by the groups EP (5.9 ± 0.67), EX (6.6 ± 1.22), and OS (6.4 ± 1.42), respectively, no change was detected in the group C scores for the determined time intervals. At T2, VAS scores continued to decrease following the same trend as at T1 time, thus there was a significant intragroup difference between T1 and T2 (*p* = 0.034).

### SSI

**Fig. 5:**
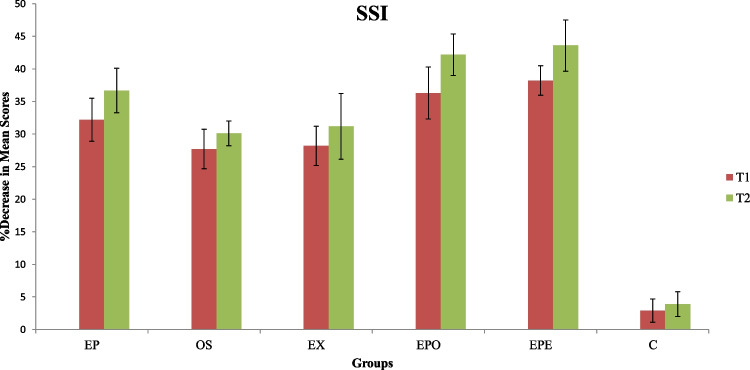
Decrease in modified Symptom Severity Index(mSSI) values of the groups (EP, Earplug group; OS, Occlusal splint group; EX, Exercise group; EPO, Earplug + Occlusal Splint, EPE, Earplug + Exercise group; C, Control group; T1,1-month follow-up; T2,3-months follow-up;*, significant difference)

All intervention groups displayed reductions in mean SSI scores from T0 to T2, reflecting improvement. As in VAS scores, the decrease at T1 and T2 was primarily seen in the combined earplug groups (groups EPE (38.2 ± 2.27, 43.6 ± 3.94) and EPO (36.3 ± 3.97, 42.2 ± 3.19)), and the differences between other groups rose to the level of statistical significance (*p* = 0.025) **(**Fig. 5). It was determined that the decrease rate in the group EP (32.2 ± 3.32, 36.7 ± 3.43) was higher than the groups EX (28.2 ± 2.99, 31.2 ± 3.03) and OS (27.7 ± 3.02, 30.1 ± 1.89) in the evaluated time intervals, respectively. There was no significant change in the values of group C during the evaluation process.

### MMO

**Fig. 6:**
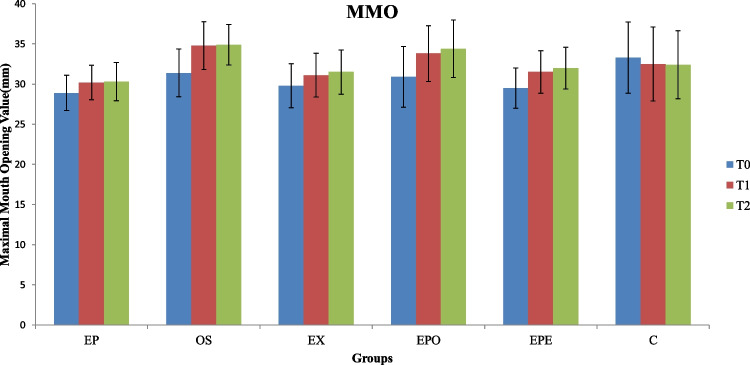
Maximum mouth opening (MMO) values of the groups (EP, Earplug group; OS, Occlusal splint group; EX, Exercise group; EPO, Earplug + Occlusal Splint, EPE, Earplug + Exercise group; C, Control group; T0,before the intervention; T1,1-month follow-up; T2,3-months follow-up;*, significant difference)

The mean and standard deviation of MMO values of the groups at T0, T1, and T2 are shown in Fig. 6**.** At T0, only the mean value of group C was higher than the other test groups (p = 0.041). At T1, the occlusal splint groups (groups OS (34.8 ± 2.97 mm) and EPO (33.8 ± 3.49 mm)) had the highest MMO values, while the lowest values were detected in group C (*p* < 0.001). At T2, all groups had similar scores with T1. Thus, there was no significant intragroup difference between T1 and T2 (*p* > 0.05). Differently, group C did not show any statistically significant increase or decrease in measured values.

## Discussion

In the present study, earplug therapy combined with the occlusal splint and exercise increased mouth opening and reduced pain and the perception of symptom severity in patients with myogenous TMD. Thus, the null hypothesis tested in this study, the first attempt at evaluating combined earplug therapy in the literature for patients with myogenous TMD, was accepted. It was also determined that earplug therapy alone affected the evaluated parameters, although it was not as effective as combined therapy models.

A growing consensus recommends conservative treatment modalities as initial therapy for patients with TMD [4]. The National Institute of Dental and Craniofacial Research noted that “experts strongly recommend using the most conservative, reversible treatments possible” to treat TMD [20]. Therefore, as a noninvasive treatment, earplug devices are considered a reversible and conservative treatment option that approaches TMD from a different perspective [6, 7]. The fact that ear devices and TMJ condyles are anatomically close to each other is accepted as the mechanism of action of these devices [11]. Although some studies evaluate the possible effect of earplugs in TMD, no study has questioned the combined effect of these devices in managing myogenous TMD patients [6, 7, 11].

There are limited studies in recent literature where earplug therapy has been applied to patients with bruxism and TMD [6, 7, 11]. These devices have been demonstrated to be a helpful therapy model through the induction of physiological effects, such as an increase in mouth opening, as well as a reduction in discomfort and muscle pain [6, 7, 11]. Earplug therapy was used in the present study based on the effect of changing the position of the mandible and thus of the condyles, reduction of the reflex activity of the musculature, and biofeedback stimulation [7, 21]. The data obtained in the groups EP, EPO, and EPE can be attributed to the close relationship between the TMJ and earplug devices, which can lead to shifts in muscle activity [6, 11, 21]. Moreover, the results obtained may also be due to the 'principle of (activity)-induced stimulation,' which is based on vibratory, mechanical, electrical, acoustic, or nociceptive stimuli and causes an immediate 'response' by providing an effect on muscle activity [11].

While the most significant decrease in VAS was seen in the EPO (5.2 ± 0.91; 3.2 ± 0.78) and EPE (5.3 ± 1.05; 3.3 ± 0.7) groups at T1 and T2, the reduction in the group EP (5.9 ± 0.67; 4.1 ± 0.63), where only the earplug was used, was significantly less than these two groups. The VAS decreases in the EX and OS groups, where splint and exercise alone were applied, were at statistically similar levels, and their effectiveness was significantly less than the group EP for both time intervals (Fig. 4). This is supported by the finding in the literature that earplugs may produce these results because they represent a more near-field approach to solving temporomandibular joint-related problems compared to the more distant-field approach of flat-plane occlusal stabilization splints [6]. Since this study was the first to evaluate the combined effect of earplugs with occlusal splints and exercise therapy, a direct comparison of the combined effect with any study in the literature could not be made. Comparison with the literature was only possible with a few studies which examined the impact of earplug treatment alone. In a previous study that preferred to compare earplug and splint therapies within three months of follow-up, it was observed that using an earplug (3.82 ± 1.83) provided a faster decrease in VAS than splints (6.16 ± 1.97) in the 1st month and gave similar results in the 3rd month [7]. In another study where splint (49%), exercise (51%), and earplug (58%) were evaluated as separate study groups and a 3-months of follow-up period was observed, it was found that the fastest VAS decline was in the earplug therapy group (p < 0.0001) [6]. In a study conducted using direct patient feedback rather than the VAS scale for PI, it was observed that earplug therapy reduced pain and tension in participants. At the same time, it did not produce the expected results in some patients [11]. Considering the limited number of studies in the literature, it can be said that the current study had comparable results with other studies regarding pain reduction, and further studies are needed. Although the follow-up period in the current study was three months, which is the same as the compared studies in the literature, it was observed that the initial and final data were different, and this could be attributed to the type and degree of the TMD disease of the patients included in the studies, the number of participants, the fabrication method of earplugs (ready-made or custom), and their material.

In addition to VAS, the mSSI index was used in the current study to measure pain in the patients by assessing any possible change in pain locations of the groups. It was observed that the results obtained from this index in question followed the same pattern as did the VAS, and the fact that the most significant decrease in the mSSI score was in the EPO (36.3 ± 3.97; 42.2 ± 3.19) and EPE (38.2 ± 2.27; 43.6 ± 3.94) groups confirmed the VAS results for both T1 and T2 times **(**Figs. 4,5). While the mSSI decrease rate in the groups was monitored by the EP, OS, EX, and control groups, respectively, no significant difference was detected between the OS and EX groups. Although it was not possible to compare the results obtained from the combined treatment groups (EPO and EPE), it was determined that the data received from the EP group were compatible with the results of the only study conducted on the subject in the literature, demonstrating the superiority of earplug use in SS [6].

Another clinically significant point about earplug use was that in groups where earplug was included, a change of location was achieved where the pain was predominantly felt by the patients based on mSSI. While the dominant pain region at T0 in the EPE and EPO groups was the 'temple,' this location was determined to be the 'masseter' at T2. Similarly, while the T0 pain region in group EPE was 'temple and masseter,' this location changed to 'masseter' at T2. While the T0 pain region was predominantly 'TMJ and masseter' in groups OS, EX, and C, this situation remained the same at T2. It may be attributed that the reason for this location change in the groups with earplugs was due to the decrease in the feeling of 'tension' resulting from the use of earplugs, which was found to be up to 70% in a previous study where earplugs were assessed, warranting further studies [12].

A statistically significant increase in MMO was observed in groups OS (34.8 ± 2.97; 35.9 ± 2.51) and EPO (33.8 ± 3.49; 34.4 ± 3.59) when compared to other groups at T1 and T2 **(**Fig. 6). The increase in MMO seen in these groups in which splint was included showed its effect in the 1st month (T1), and the increasing trend continued in the 3rd month (T2). The present study's findings agreed with the study from Lange and Peroz, which reported a similar MMO increase in patients with TMD and bruxism who used earplugs [12]. However, the rise in MMO seen at T1 in the OS group in this study was observed only in the earplug group from another study, and it was determined it could not be compared to the current study [7]. This discrepancy can be due to the fabrication method of earplugs and splints, daily use time of these devices, patients' compliance, type, and degree of TMD of the participants included. Thus, the possible clinical effects of the earplugs should be evaluated in future studies with standardized clinical set-ups.

The effectiveness of exercise therapy and occlusal splint was equivalent in VAS and SSI. At the same time, it was statistically determined that splint was superior to exercise in terms of MMO for both T1 and T2 in this study. While a recent systematic review with meta-analysis supported the exercise’s disinhibiting effect on pain in VAS, the impact of exercise in MMO contributed to the controversial results in the literature [22]. While a group of studies in the literature argued that exercise therapy positively improved MMO, some reported that it had the same effect as splints [22, 23]. Considering that the occlusal splint corrects muscle disequilibrium and maintains TMJ in functional posture free of pain and the effect of exercise therapy on increasing resistance and strength of masticatory muscles, it is understood that the results obtained from this study have a clinical impact [22]. Another point that should be mentioned is that combining these two types of treatment with an earplug produced a positive effect for PI and SS but not for MMO.

The ability to apply TMD treatments in combination with an earplug varies depending on the patient's ability to use this device and his/her compliance with the treatment. Two patients were excluded from the present study because they reported feeling pressure when using these earplugs at night. For these earplugs to be easily used by patients, the patient's ability to tolerate such devices and their motor skills should be evaluated by the dental practitioner at the beginning of the treatment. Another possible factor that complicates using these earplugs is individual differences in ear anatomy, and the patient should be given a 'trial period' before these devices are included in the treatment, as applied in this study.

The limitations of the current study include the short span for follow-up, lack of blinding, the predominance of female patients, the sole consideration of myogenous TMD, and the lack of characterization of the degree of disease. The sample consisted mainly of female patients, consistent with the literature knowledge that females are more affected by TMD and seek more treatment than males. Further studies with more extended evaluation periods and larger sample sizes are needed to verify the clinical evidence of earplug therapy in patients with different types of TMD. Another point that should be evaluated in future studies may be the effect of 'triple therapy' combinations in which the earplug will be included (such as splint-earplug-exercise).

## Conclusions

Short-term use of earplugs combined with occlusal splint and exercise therapies produced significantly lower PI, SS, and an increased MMO. While the application of earplug therapy alone gave more positive results than splint and exercise therapies in VAS and mSSI indices, the opposite trend was observed in MMO scores after one to three months of use in patients with myogenous TMD. These devices can be suggested as a complementary therapy to widely used TMD management, such as exercise and splint therapy. Therefore, considering the high prevalence of myogenous TMD, this treatment method should be considered by clinicians as an option due to its accessibility, safety, effectiveness, low complication rate, and cost-effectiveness.

## Data Availability

No datasets were generated or analysed during the current study.

## References

[1] Pierson MJ (2011) Changes in temporomandibular joint dysfunction symptoms following massage therapy: a case report. Int J Ther Massage Bodywork 4:37–47. 10.3822/ijtmb.v4i4.110 22211156 10.3822/ijtmb.v4i4.110 PMC3242647

[2] Benli M, Eker Gümüş B, Kahraman Y, Gökçen-Rohlig B, Evlioğlu G, Huck O, Özcan M (2020) Surface roughness and wear behavior of occlusal splint materials made of contemporary and high-performance polymers. Odontology 108:240–250. 10.1007/s10266-019-00463-1 31612354 10.1007/s10266-019-00463-1

[3] McKinney MW, Londeen TF, Turner SP, Levitt SR (1990) Chronic TM disorder and non-TM disorder pain: a comparison of behavioral and psychological characteristics. Cranio 8:40–46. 10.1080/08869634.1990.11678298 2098186 10.1080/08869634.1990.11678298

[4] Benli M, Olson J, Huck O, Özcan M (2023) A novel treatment modality for myogenous temporomandibular disorders using aromatherapy massage with lavender oil: A randomized controlled clinical trial. Cranio 41:48–58. 10.1080/08869634.2020.1819067 32893748 10.1080/08869634.2020.1819067

[5] Kalamir A, Graham PL, Vitiello AL, Bonello R, Pollard H (2013) Intra-oral myofascial therapy versus education and self-care in the treatment of chronic, myogenous temporomandibular disorder: a randomised, clinical trial. Chiropr Man Therap 5(21):17. 10.1186/2045-709X-21-17 10.1186/2045-709X-21-17 PMC3706243 23738586

[6] Tavera AT, Montoya MC, Calderón EF, Gorodezky G, Wixtrom RN (2012) Approaching temporomandibular disorders from a new direction: a randomized controlled clinical trial of the TMDes ear system. Cranio 30:172–182. 10.1179/crn.2012.027 22916669 10.1179/crn.2012.027

[7] Mowafy MI, Abdella AA (2021) The use of therapeutic ear plugs for treatment of myogenous TMD: a randomized controlled clinical trial. Egy Orth J 60:86–97. 10.21608/eos.2021.113048.1039

[8] Magdaleno F, Ginestal E (2010) Side effects of stabilization occlusal splints: a report of three cases and literature review. Cranio 28:128–135. 10.1179/crn.2010.018 20491235 10.1179/crn.2010.018

[9] Tuz HH, Onder EM, Kisnisci RS (2003) Prevalence of otologic complaints in patients with temporomandibular disorder. Am J Orthod Dentofacial Orthop 123:620–623. 10.1016/s0889-5406(03)00153-7 12806339 10.1016/s0889-5406(03)00153-7

[10] Yu JF, Hsien HC, Lee KC, Xiao JH, Chiu HH, Hong HH, Shen YF, Peng YC (2018) Effect of mouth-opening levels on sound field gain in the ear canal. J Acoust Soc Am 143:1451. 10.1121/1.5026692 29604713 10.1121/1.5026692

[11] Lange M, Peroz I (2019) The use of ear inserts for the treatment of temporomandibular dysfunctions and bruxism. Zeitschrift für Kraniomandibuläre Funktion 11:353–369

[12] Fricton JR, Schiffman EL (1987) The craniomandibular index: validity. J Prosthet Dent 58:222–228. 10.1016/0022-3913(87)90181-8 3476731 10.1016/0022-3913(87)90181-8

[13] Juszczak E, Altman DG, Hopewell S, Schulz K (2019) Reporting of multi-arm parallel-group randomized trials: extension of the CONSORT 2010 statement. JAMA 321:1610–1620. 10.1001/jama.2019.3087 31012939 10.1001/jama.2019.3087

[14] Polat S, Polat N, Cetioglu A, Saleh SM, Unal S, Yolcu U, Tatatr T [Diagnostic Criteria for Temporomandibular Disorders: assessment Instruments] (Turkish) (2016). Temporomandibuler Düzensizlikler için Teşhis Kriterleri: değerlendirme Araçları Turkish translation by: Temporomandibuler Düzensizlikler için Teşhis Kriterleri: değerlendirme Araçları. www.rdctmdinternational.org. Accessed 2 Apr 2023

[15] Research Randomizer. https://www.randomizer.org/. Accessed April 2, 2023

[16] Okeson JP (2019) Management of temporomandibular disorders and occlusion, 8th edn. Elsevier Mosby Inc, St. Louis, Missouri, pp 385–410

[17] Capellini VK, de Souza GS, de Faria CR (2006) Massage therapy in the management of myogenic TMD: a pilot study. J Appl Oral Sci 14:21–26. 10.1590/s1678-77572006000100005 19089025 10.1590/S1678-77572006000100005 PMC4327166

[18] Nixdorf DR, John MT, Wall MM, Fricton JR, Schiffman EL (2010) Psychometric properties of the modified Symptom Severity Index (SSI). J Oral Rehabil 37:11–20. 10.1111/j.1365-2842.2009.02017.x 19889036 10.1111/j.1365-2842.2009.02017.x PMC2858780

[19] Benli M, Özcan M (2023) Short-term effect of material type and thickness of occlusal splints on maximum bite force and sleep quality in patients with sleep bruxism: a randomized controlled clinical trial. Clin Oral Investig 27:4313–4322. 10.1007/s00784-023-05049-4 10.1007/s00784-023-05049-4 37127807

[20] National Institute of Dental and Craniofacial Research (NIDCR) (2006) TMJ Disorders. NIH Publication No. 06–3487. National Institutes of Health, Bethesda, pp 9–13

[21] Imhoff B, Weber D (2019) New treatment approaches for temporomandibular dysfunction patients. J Craniomandib Func 11:141–150

[22] Idáñez-Robles AM, Obrero-Gaitán E, Lomas-Vega R, Osuna-Pérez MC, Cortés-Pérez I, Zagalaz-Anula N (2023) Exercise therapy improves pain and mouth opening in temporomandibular disorders: A systematic review with meta-analysis. Clin Rehabil 37:443–461. 10.1177/02692155221133523 36263523 10.1177/02692155221133523

[23] Zhang L, Xu L, Wu D, Yu C, Fan S, Cai B (2021) Effectiveness of exercise therapy versus occlusal splint therapy for the treatment of painful temporomandibular disorders: a systematic review and meta-analysis. Ann Palliat Med 10:6122–6132 10.21037/apm-21-451 33977737 10.21037/apm-21-451

